# Phylogenetic analysis based on the complete mitochondrial genome of *Discogobio brachyphysallidos* (Cypriniformes: Cyprinidae) suggests the need for taxonomic revision at the genus level

**DOI:** 10.1080/23802359.2024.2306882

**Published:** 2024-01-26

**Authors:** Fangcan Chen, Pingke Lu, Dejin Liang, Yuli Wu, Zhiyong Jiang, Wei Huang, Liuling Gao

**Affiliations:** a Guangdong Hanyu Ecological Technology Co., Ltd., Guangzhou, China; b Guangdong Tilapia Breeding Farms, Guangzhou, China; c Agro-Tech Extension Center of Guangdong Province, Guangzhou, China

**Keywords:** Mitochondrial genome, *Discogobio brachyphysallidos*, phylogenetic relationships, genus *Discogobio*

## Abstract

*Discogobio brachyphysallidos* Huang 1989 is a Cyprinidae fish species that is endemic to the upper Pearl River. In the present study, the complete mitochondrial genome of *D. brachyphysallidos* collected from the Nanpanjiang River was sequenced and annotated. The mitochondrial genome encompassed 13 protein-coding genes, two ribosomal RNA (rRNA) genes, 22 transfer RNA (tRNA) genes, and the control region (D-loop). The total length of the mitochondrial genome was determined to be 16,594 base pairs (bp), with a GC content of 41.7%. Phylogenetic analyses revealed that *D. brachyphysallidos* may be a sister to *D. longibarbatus* and *D. macrophysallidos*. These findings provide insight into the genetic information and phylogenetic relationships of *D. brachyphysallidos*.

## Introduction

**Figure 1.**
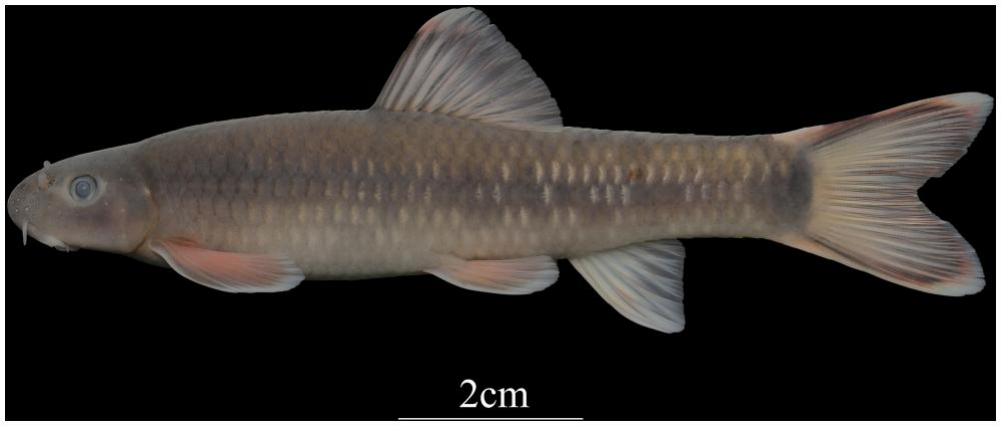
Photograph of *Discogobio brachyphysallidos* (by Jiahu Lan).

**Figure 2.**
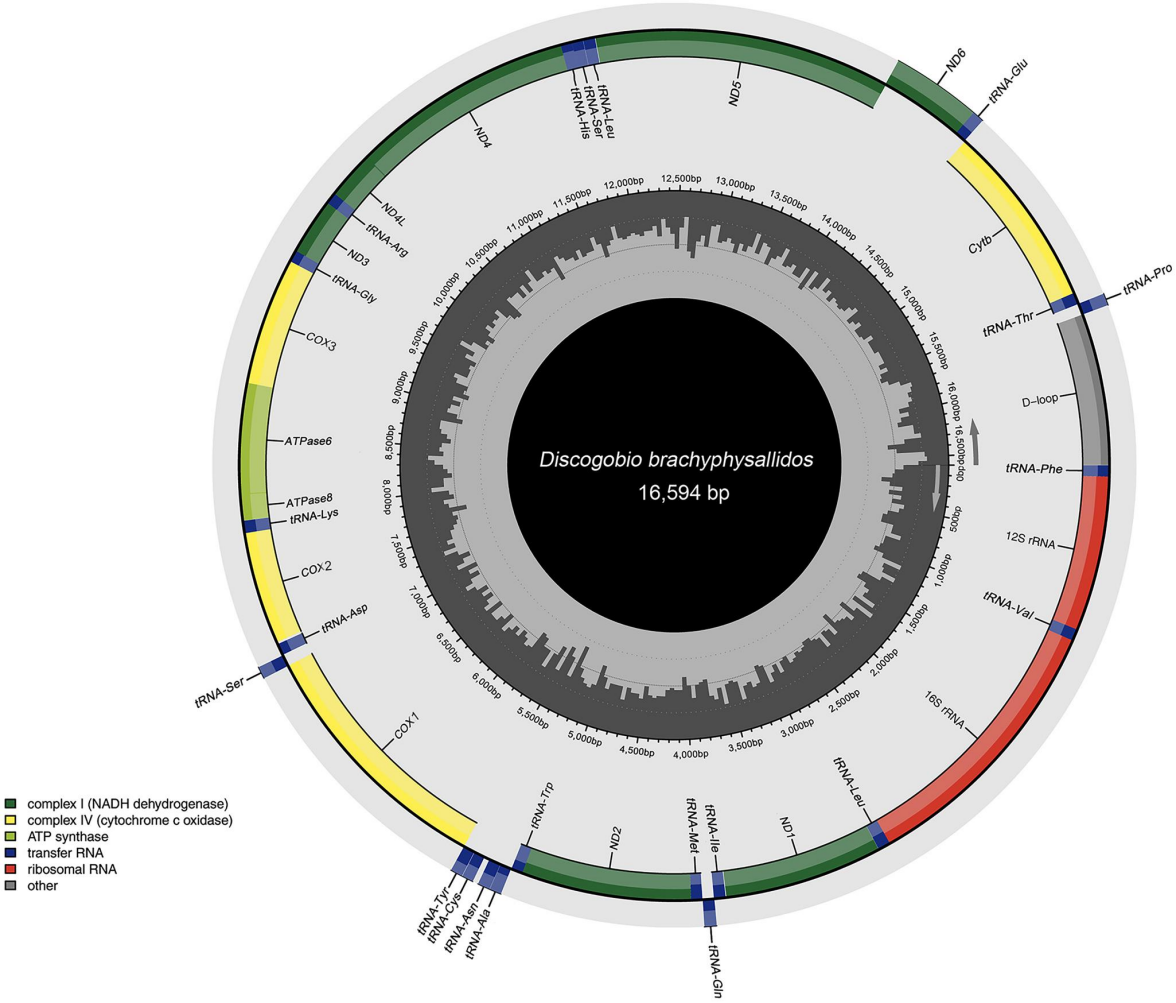
Genome map of *Discogobio brachyphysallidos* mitochondrial genome. The inner and external circles represent the GC content and the genes having different colors based on their functions, respectively. The arrows represent direction of transcription. The genes encoded on the heavy and light strand are shown outside and inside the circle, respectively.

The genus *Discogobio* comprises a benthic fish group in southern China and falls under the subfamily Labeoninae. According to the FishBase dataset (www.fishbase.org), seventeen recognized species are documented in this genus. *Discogobio brachyphysallidos* Huang 1989 was found to be distributed primarily within the Nanpanjiang River and upper Youjiang River and can be distinguished from all other congeners by a combination of the following characteristics: small swim bladder, lack of a snout, undivided bead star on the rhynchodaenm and large suction cup (Huang 1989; Xiao and Lan 2023). Recent phylogenetic investigations have shed light on the phylogenetic relationships within this genus using four nuclear loci (Zheng et al. 2012). These studies indicated that *D. brachyphysallidos* was initially grouped with *D. macrophysallidos* (Zheng et al. 2012). Given that only nuclear markers were used in this study, to better understand the phylogenetic placement of this species, it is highly necessary to clarify its phylogenetic status using matrilineal loci, such as the mitogenome. In the present study, we sequenced and annotated the complete mitogenome of *D. brachyphysallidos* and inferred its phylogenetic placement (Figures 1 and 2).

## Materials

One specimen of *D. brachyphysallidos* was caught on July 31, 2022, from Lubuge Town, Luoping County, Yunnan Province, China (24.749764°N, 104.509222°E; elevation: 786 m) and was identified using a combination of characteristics, including a small swim bladder, lack of a snout, an undivided bead star on the rhynchodaenm and a large suction cup. This specimen was stored at Guangdong Hanyu Ecological Technology Co., Ltd., Guangzhou City, China (sample ID: DPPJ-QJ01, www.gdhystkj.com; contact person: Fangcan Chen; email: cfc3210@163.com).

## Methods

Genomic DNA was extracted utilizing a Genomic DNA Isolation Kit (Qiagen, Hilden, Germany). Library pooling and whole-genome sequencing of *D. brachyphysallidos* were performed using the Illumina MiSeq platform (Illumina, San Diego, CA) with 150 bp paired-end reads. A total of 8.08 × 10^9^ bp of raw data were generated and filtered in SOAPnuke 1.3 (Chen et al. 2018). We assembled the mitogenome using MITOZ (Meng et al. 2019). The sequence depth and coverage of the mitogenome were generated following the protocol of Ni et al. (2023). A map of the coverage depth was generated, and the average depth was approximately 469 × (Supplementary Materials, Figure S1). The assembled mitogenome was annotated, and a map of the mitogenome was generated using the online tool MitoAnnotator (Iwasaki et al. 2013; Zheng et al. 2020). Published mitogenomes containing five *Discogobio* species (*D. longibarbatus* KY465995 (Zheng and Yang 2017a), *D. macrophysallidos* MT536775 (Qiu et al. 2020), *D. yunnanensis* KJ997760 (Xue et al. 2016), *D. laticeps* MH127917 (Tan et al. 2019), and *D. tetrabarbatus* KJ669372 (Zhao et al. 2016b)), two *Discocheilus* species (*D. wuluoheensis* KX840359 (Zheng and Yang 2017b) and *D. wui* KX840358 (Zheng and Yang 2017b)), and an outgroup, *Ptychidio jordani* (GenBank accession: KJ620837; Zhao et al. 2016a), were downloaded from GenBank for phylogenetic analyses. Furthermore, two *Discocheilus* species were included in the phylogenetic analyses because they were found to be phylogenetically placed within the genus *Discogobio* in previous studies (Zheng et al. 2012; Zheng and Yang 2017a; Zheng and Yang 2017b).

Overall, all the mitochondrial genomes included in the analysis were aligned using MUSCLE (Edgar 2004). Interspecific genetic distance based on the Kimura 2-parameter model (Kimura 1980) was calculated using MEGA X (Kumar et al. 2018). Maximum likelihood (ML) analysis was performed with RAxML-VI-HPC (Stamatakis 2006) using the GAMMA model and 1000 bootstrap replicates. Bayesian inference (BI) analysis was conducted in MrBayes 3.2 (Ronquist et al. 2012) with four Markov chain Monte Carlo (MCMC) chains using the following parameters each: GTR substitution model (nst = 6) with gamma-distributed rate variation (rates = invgamma) across sites and a proportion of invariable sites (statefreqpr = dirichlet (1,1,1,1)). These parameters were selected by MrModeltest (Nylander 2004). This run was performed for 20 million generations, with sampling every 1000 generations. The first 25% of the generations were discarded as burn-in.

## Results

The mitochondrial genome of *D. brachyphysallidos* was 16,594 bp in length, encoding 13 protein-coding genes (PCGs), two ribosomal RNA (rRNA) genes, and 22 transfer RNA (tRNA) genes. In addition, the mitogenome also contained a 934 bp long putative control region (D-loop). The nucleotide composition was 32.1% A, 26.2% T, 15.4% G, and 26.3% C. Among the genes, *ND6*, *tRNA-Glu*, *tRNA-Pro*, *tRNA-Gln*, *tRNA-Ala*, *tRNA-Asn*, *tRNA-Cys*, *tRNA-Tyr*, and *tRNA-Ser* ^(CGA)^ were on the L-strand, while the rest were on the H-strand. Most PCGs start with ATG codons, except for *COX1* with GTG. The stop codons vary; TAA is used by *ND1*, *COX1*, *ATP6*, *APT8*, *ND4L*, *ND5*, and *ND6*; T is used by *ND2*, *COX2 ND3*, *ND4*, and *Cytb*; and TA is used by *COX3*.

**Figure 3.**
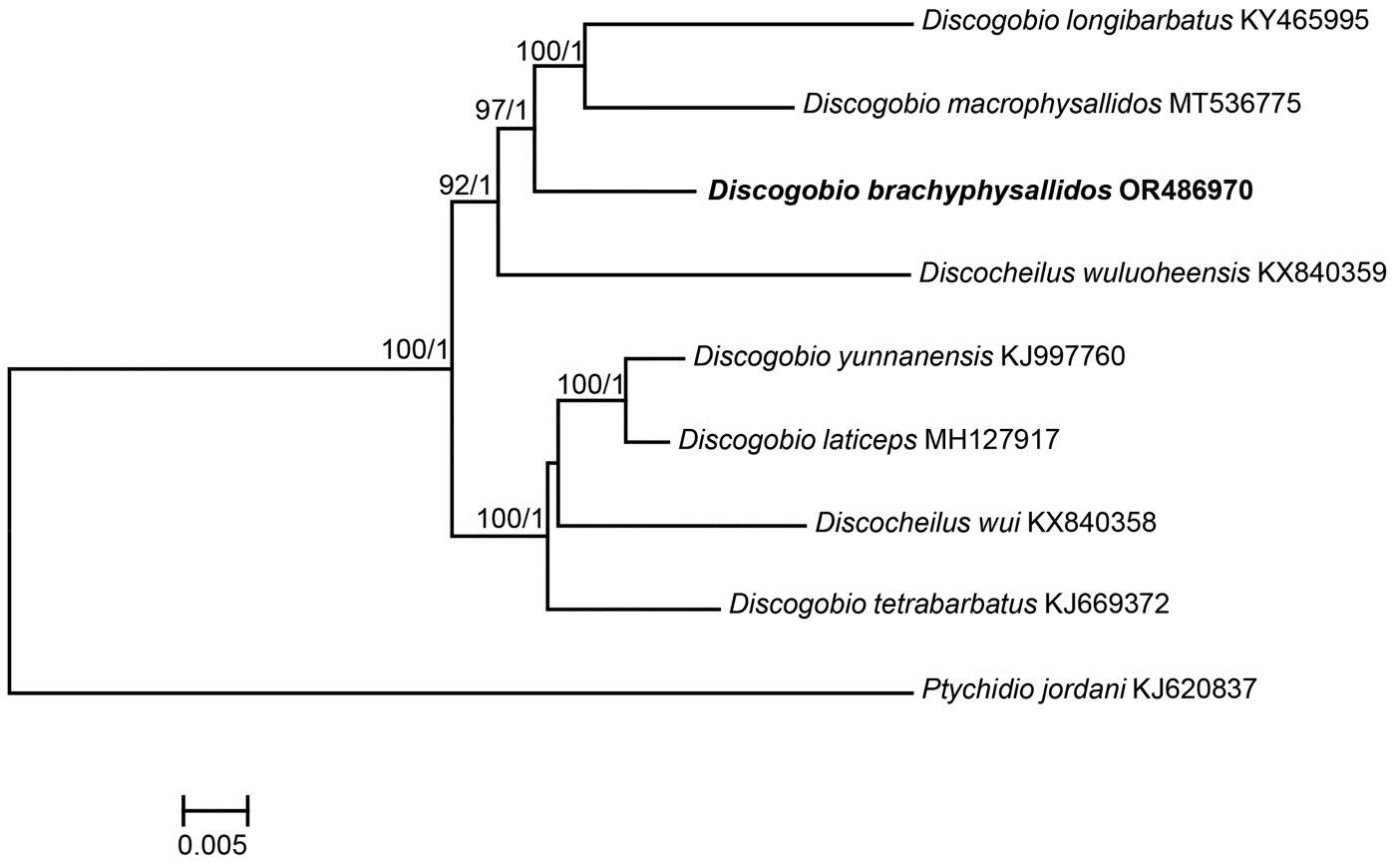
Maximum likelihood tree based on the mitochondrial genome. The values on the branches represent bootstrap proportions derived from a maximum likelihood analysis and Bayesian posterior probabilities, respectively. The sample under investigation is highlighted in bold.

ML and BI trees produced similar topologies, which indicated that *D. brachyphysallidos* was sister to the clade that includes *D. longibarbatus* and *D. macrophysallidos* (Figure 3). In addition, the trees showed that the two *Discocheilus* species were grouped with the *Discogobio* species (Figure 3). The genetic distance between *D. brachyphysallidos* and the other five *Discogobio* species ranged from 0.0284 to 0.0342.

## Discussion and conclusion

The gene order and composition were identical to those of other *Discogobio* species (Xue et al. 2016; Zhao et al. 2016a; Zhao et al. 2016b; Zheng and Yang 2017a; Zheng and Yang 2017b; Tan et al. 2019; Qiu et al. 2020). Phylogenetic trees revealed the close relationship between *D. brachyphysallidos* and the clade comprising *D. longibarbatus* and *D. macrophysallidos*, which was in line with the conclusions drawn from the analysis of nuclear loci (Zheng et al. 2012). Moreover, *Discogobio* and *Discocheilus* clustered together, suggesting that these two genera are not distinct. These genera did not exhibit reciprocal monophyly in the tree. Considering that limited taxonomic coverage and molecular loci were used in the present study, the phylogenetic relationships among *Discogobio* species may not be robust, and further studies involving larger taxon sampling and additional nuclear loci are necessary.

## Supplementary Material

Supplemental Material

## Data Availability

The mitogenome sequence data are available in the GenBank of NCBI at https://www.ncbi.nlm.nih.gov/ under the Accession No. OR486970. The associated BioProject, SRA, and Bio-Sample numbers are: PRJNA1008996, SRR25755686, and SAMN37141522, respectively.
