# Supplementary material for: Phylogenetic analysis based on the complete mitochondrial genome of Discogobio brachyphysallidos (Cypriniformes: Cyprinidae) suggests the need for taxonomic revision at the genus level
Source: Mitochondrial DNA B Resour. 2024 Jan 26;9(1):200–3. doi: 10.1080/23802359.2024.2306882 (PMC10823891; doi:10.1080/23802359.2024.2306882)
Supplement: Supplemental Material [file TMDN_A_2306882_SM2974.docx]

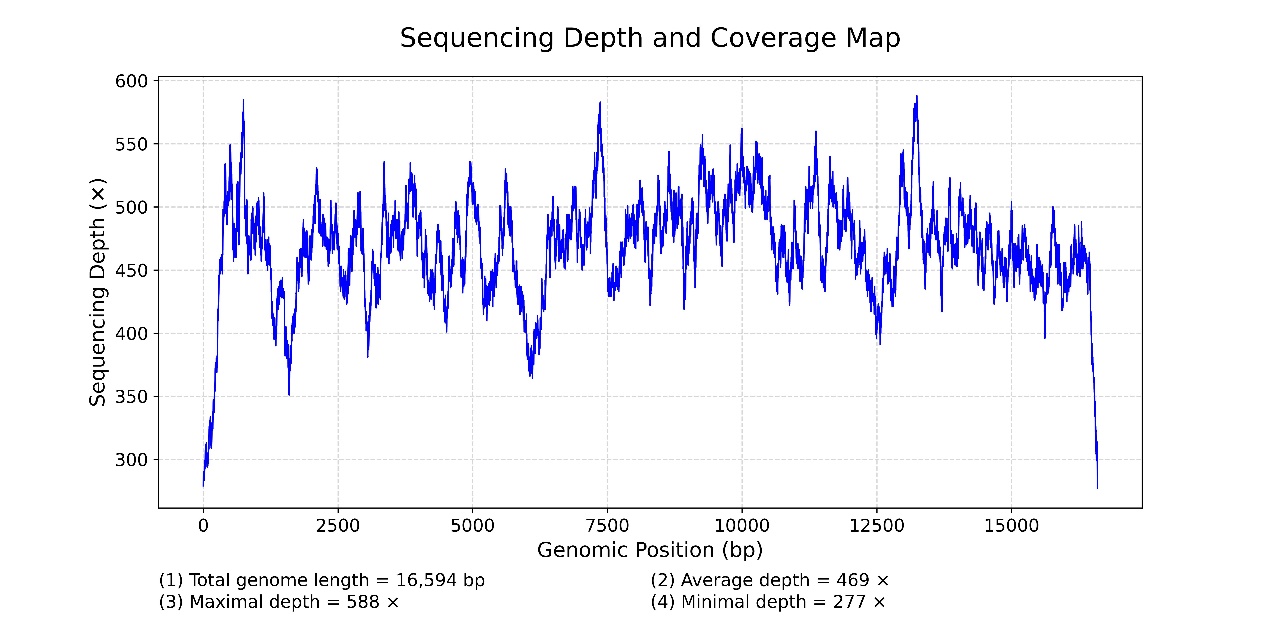


**Figure S1** Generating Sequencing Depth and Coverage Map for *Discogobio brachyphysallidos* mitochondrial genome. X and Y axis present nucleotide position of *D. brachyphysallidos* mitochondrial genome and read mapping depth, respectively.
